# Postprandial Satiety, Glycemia, and Antioxidant Capacity of Apparently Healthy Adults After Consuming Corn Chips with Avocado Paste Byproduct

**DOI:** 10.3390/foods15193359

**Published:** 2026-09-22

**Authors:** B. Shain Zuñiga-Martínez, J. Abraham Domínguez-Avila, Rosario Maribel Robles-Sánchez, J. Fernando Ayala-Zavala, Marcelino Montiel-Herrera, Norma Julieta Salazar-López, Manuel Viuda-Martos, Mónica A. Villegas-Ochoa, Gustavo A. González-Aguilar

**Affiliations:** 1 Centro de Investigación en Alimentación y Desarrollo A. C., Carretera Gustavo Enrique Astiazarán Rosas No. 46, Col. La Victoria, Hermosillo 83304, Sonora, Mexico; shain.zuniga21@gmail.com (B.S.Z.-M.); jayala@ciad.mx (J.F.A.-Z.); mvillegas@ciad.mx (M.A.V.-O.); gustavo@ciad.mx (G.A.G.-A.); 2 SECIHTI-Centro de Investigación en Alimentación y Desarrollo A. C., Carretera Gustavo Enrique Astiazarán Rosas No. 46, Col. La Victoria, Hermosillo 83304, Sonora, Mexico; 3 Departamento de Investigación y Posgrado en Alimentos, Universidad de Sonora, Blvd. Luis Donaldo Colosio y Rosales S/N, Col. Centro, Hermosillo 83000, Sonora, Mexico; maribel.roblessanchez@unison.mx; 4 Departamento de Medicina y Ciencias de la Salud, Universidad de Sonora, Blvd. Luis Encinas y Rosales S/N, Col. Centro, Hermosillo 83000, Sonora, Mexico; marcelino.montiel@unison.mx; 5 Facultad de Medicina y Nutrición de Mexicali, Universidad Autónoma de Baja California, Dr. Humberto Torres Sanginés, Centro Cívico, Mexicali 21000, Baja California, Mexico; norma.salazar@uabc.edu.mx; 6 IPOA Research Group, Instituto de Investigación e Innovación Agroalimentaria y Agroambiental (CIAGRO-UMH), Universidad Miguel Hernández, 03312 Alicante, Spain; mviuda@umh.es

**Keywords:** enteric hormones, oxidative stress, serum glucose

## Abstract

Avocado paste (AP) is the main byproduct of industrial avocado processing. It has been recently studied as a source of potentially bioactive compounds with demonstrated in vivo satiety-related actions; however, these have not been explored in human consumers. The present study analyzed the effects of consuming AP-supplemented corn chips (AP-10%) on postprandial glycemia (0, 15, 30, 60, 90, and 120 min post-consumption). Subjective satiety was also analyzed during the aforementioned times, while serum satiety mediators (insulin, glucagon-like peptide-1 [GLP-1], and cholecystokinin [CCK]) and antioxidant capacity were quantified (0 and 120 min post-consumption) in eighteen apparently healthy adult consumers. Maximum glycemia occurred 15 min after consuming the chips (137 mg/dL), which the AP-supplemented samples were able to significantly decrease (118 mg/dL). Subjective satiety was positively affected, as determined by visual analogue scales, which showed that AP-supplemented samples increased the participants’ sensations of satiety and fullness by approximately 28 and 29%, respectively; trends were observed on the concentration of serum satiety mediators, although they were not significant. AP supplementation also mitigated a decrease in postprandial antioxidant capacity, as compared to the control, which showed a significantly lower value, according to the ORAC assay. We conclude that AP-supplemented corn chips may have an impact on postprandial glycemia, subjective satiety, and antioxidant capacity of apparently healthy adult consumers. Further studies are required to determine and/or complement the health effects of prolonged consumption of AP or AP-supplemented products.

## 1. Introduction

Mexico is the main producer, exporter, and consumer of avocado (*Persea americana* Mill.) [1]. Its fruits are well-accepted and consumed raw due to their organoleptic properties, but they are also used to obtain other products like avocado oil, due to their high lipid content [2]. Oil extraction can be done by different methods, although peel, seed, and defatted pulp are the main byproducts of all of them. These may be produced separately or combined into a homogeneous mixture known as avocado paste (AP).

The use of vegetable-derived byproducts, including AP, has been a focus point of many recent studies, whose aim is to make integral use of agricultural products while minimizing wasted material [3]. If a given byproduct contains nutrients and/or bioactive compounds that can be used within the food industry, validating their in vitro or in vivo effects can contribute to supporting their use. Avocado consumption has been linked to multiple health benefits, including maintaining a healthy weight [4], and normalizing glycemia and the serum lipid profile [5], among other effects, mainly due to its fatty acid and fiber content. In contrast, avocado byproducts have been studied only recently, for example, potential in silico interactions between AP-derived phenolics (such as protocatechuic acid, ferulic acid, and *p*-coumaric acid) may contribute to their cholesterol-lowering effects [6], while decreasing hunger and inducing satiety in an in vivo model [7]. Even if the evidence is promising, avocado byproducts have only been studied in silico and in vivo, while human data is currently scarce and merits further study.

The hunger/satiety sensations are crucial to maintain health by regulating ingested energy, which must be balanced with expended energy [8]. These sensations are part of a complex and multifactorial system, where multiple intrinsic (such as hormonal) and extrinsic (such as time of day) variables converge to regulate an individual’s perception of the immediate need and desire to ingest food [9]. Thus, when analyzing the effects of a given food, substance, or stimulus on the hunger/satiety system, some variables are inherently subjective, such as the perception of hunger, while others are objective, such as the serum concentration of a given biomarker. The effect of a food on postprandial glycemia and insulinemia may be associated with its effects on hunger/satiety [10]. The postprandial serum concentration of glucagon-like peptide-1 (GLP-1) and cholecystokinin (CCK) is also relevant, since these are two other important enteroendocrine biomarkers associated with hunger/satiety responses [11], although many others are known to play a role [12]. Moreover, the glycemic impact of a food is also crucial for its health effects beyond hunger/satiety, since those with a high effect tend to be less desirable, as compared to those with a medium or low effect [13].

Corn chips are a suitable matrix to which a potentially functional additional ingredient can be added, in order to determine its postprandial effects. For example, they are a simple food matrix formed by a limited number of well-known ingredients, which allows precise control over their composition. They are commonly consumed in Mexico (where the present study was performed), as well as in multiple other places around the world, allowing for simple replicability of the study. Previous studies report that adding AP to them is viable [14] and, given their carbohydrate-rich nature, they can be used to analyze the impact of AP on carbohydrate digestion and postprandial glycemia.

Previous experiments have shown that long-term consumption of AP phenolics (eight weeks) has a significant effect on the hunger/satiety responses of rats, according to increased satiety; such effects were attributed to changes in the serum concentration and gene expression of relevant hormones (GLP-1, leptin, and adiponectin) [7]. Given the acceptability of AP-supplemented chips by potential consumers [14], the present experiment aims to use AP as an ingredient in the preparation of corn chips, and to identify the postprandial (acute) effects of their consumption regarding the hunger/satiety-related responses of healthy human consumers. To the best of our knowledge, this has not been previously reported.

## 2. Materials and Methods

### 2.1. Samples

AP was donated by an avocado-processing plant in the state of Michoacán, one of the main avocado-producing regions in Mexico. The samples used consisted of defatted avocado pulp, and ground peel and seed in unspecified proportions. The samples were received at the laboratory, and immediately frozen and freeze-dried at −50 °C (FreeZone 6 Liter Benchtop, Labconco Corporation, Kansas, MO, USA). Freeze-dried AP was sieved in order to homogenize its particle size (<250 µm mesh), and stored in amber bags at −20 °C until used to prepare corn chips as previously described [14]. In brief, a control chip was prepared, which contained 100% commercially available corn flour, while an AP-enriched sample contained 90% corn flour and 10% AP, and was referred to as AP-10%. The dough of each sample was hand-kneaded, pressed with a manual press, and cut into thin triangles. The obtained pieces were preheated in an electric pan and baked in an oven for 7 min at 180 °C. Samples were cooled to room temperature and stored in amber bags until they were administered. The sensory properties and proximate composition of the chips have been previously reported; the control chips contain 83.79% carbohydrates, 2.72% fiber, 2.98% lipids, and 7.76% protein, while the AP-enriched samples contain 77.16% carbohydrates, 5.29% fiber, 3.62% lipids, and 7.75% protein [14]. The phenolic profile of the AP and AP-enriched samples used is also known, and consists of 17 different compounds classified within seven chemical families, namely, flavonols, phenolic acids, flavones, flavanones, lignans, phenolic aldehydes, and anthocyanins, with pinoresinol, quercetin-3-O-glucoside, hesperidin, quercetin-3-O-glucuronide, and ferulic acid being the five most abundant species [15].

A 10% supplementation level was chosen according to previous experiments [14,15], where AP-supplemented corn chips were analyzed regarding their nutritional composition. A 10% supplementation was shown to contain the highest content of multiple phenolic compounds, and was therefore chosen to be investigated in the present study.

### 2.2. Participants

All experimentation that involved human participants was first submitted to the Research Ethics Committee of CIAD, which reviewed and approved the protocol (CEI/032-1/2021, 3 February 2022). Experiments followed all applicable national and international regulations, as well as the Declaration of Helsinki.

Participants were recruited via social media, flyers, and in-person invitations. Inclusion criteria were being 18–29 years old, having a normal weight according to a body mass index (BMI) of 18.5–24.9, having a fasting glycemia of 70–100 mg/dL, and being in good health overall. Exclusion criteria were having non-communicable diseases that could impact their hunger/satiety sensations (such as diabetes, cancer, gastrointestinal diseases, or thyroid diseases, among others), being pregnant or breastfeeding, being under pharmacological intervention or having undergone surgery within the past 6 months, being smokers, and consuming antioxidant or probiotic supplements. Sixteen apparently healthy women and two men (eighteen total) fulfilled these criteria and were willing to take part in the study.

The participants were interviewed in order to corroborate that they met all the inclusion criteria. The purpose of the study was then explained to them, in addition to what their participation would entail regarding the time required, consuming corn chips, answering hunger/satiety questions, and having capillary and venous blood drawn on two different days, separated by one week. No compensation of any kind was offered to them in exchange for their participation; they were also informed that they could withdraw from the study at any point and for any reason, and that their answers and variables analyzed from their blood were going to be anonymously published in a scientific paper. After this was explained to them and they agreed to participate, they signed an informed consent form; their height and weight were then measured (OMRON HBF-514C, Yangzhou, China) and were scheduled to participate within approximately one week.

### 2.3. Study Design

To the best of our knowledge, there are no experiments that have determined the postprandial effects of avocado byproduct consumption in healthy human beings; thus, the present experiment was designed as a pilot study meant to provide a first approach to their consumption over a short period of time (120 min).

The study took place on two days, separated by one week. The experiment was scheduled to start at 08:00 on the first day, and participants were therefore asked to arrive at the laboratory at approximately 07:45 after a fast of at least 8 h. They were also asked to maintain their regular diet and routine the day before, not to consume alcohol, caffeine, or phenolic-rich supplements, not to perform any non-routine activities that could impact their hunger on that day, and to sleep as they normally would. They were reminded of these instructions the day before the analysis via a text message, while a 24 h dietary recall was performed on the day of the analysis to verify compliance.

By approximately 08:00, the participants were seated in a comfortable climate-controlled room (approximately 24 °C). Before they consumed the corn chips, a vial of venous blood was drawn in a serum-separating tube, in addition to a drop of capillary blood in order to determine their glycemia. They were also asked to fill out a visual analogue scale (VAS) in order to establish their baseline hunger/satiety (see the following section). After all the basal measurements were collected, the participants consumed the control corn chips; they were served a portion that consisted of 50 g of total carbohydrates (59.7 g of chips; 234.6 kcal [14]), and were instructed to consume it in approximately 10 min. A 350 mL glass of water was also administered alongside the corn chips. The VAS was filled in and capillary blood was sampled 15, 30, 60, 90, and 120 min after consuming the chips. After 120 min, a second tube of venous blood was also drawn, and the study concluded for that day.

The study was exploratory and followed a crossover design, where the participants first consumed the control sample, and were asked to come back after one week to consume 50 g of total carbohydrates of the AP-supplemented one (64.8 g of chips; 241.2 kcal [14]). No additional washout period was established, nor were the participants asked to refrain from consuming a healthy diet that contained phenolic- and/or fiber-rich foods (fruits, vegetables, grains, etc.), they were only given similar instructions as those of the initial visit (to maintain their regular diet and routine the day before, not to consume alcohol, caffeine, or phenolic-rich supplements, not to perform any non-routine activities that could impact their hunger on that day, and to sleep as they normally would). The participants were not told which sample was consumed each day, in order to avoid skewing their responses.

The use of capillary blood to determine glycemia in the present study was chosen as a less invasive option than venous blood sampling. This was an effort to minimize the participants’ discomfort by limiting venipunctures to only two, one at the beginning and one at the end of the experiment.

Figure 1 shows a timeline of the variables analyzed at each sampling time.

It should be noted that all samples administered to the participants were prepared under strict hygienic standards; microbiological analyses were performed prior to administering them to the participants, and the samples were deemed safe for human consumption.

### 2.4. Subjective Hunger/Satiety Analyses

The participants’ subjective hunger and satiety sensations were analyzed through a VAS for four different questions that were answered at regular intervals during the experimental period (Figure 1). They were asked to rate their current hunger (“How hungry do you feel right now?”), prospective food consumption (“How much food do you think you could eat right now?”), satiety (“How satiated are you right now?”), and fullness (“How full do you feel right now?”). They gave their answers by making a mark on a 10 cm scale anchored with the terms “not at all” and “more than ever” at 0 and 10 cm, respectively [16,17]. They were asked to answer independently (they could not consult or discuss their answers with other participants) and were not allowed to check their own answers for previous times. For data analysis, the distances from the zero point of the scale were carefully measured and graphed; the area under the curve (AUC) of each question was then calculated using the trapezoidal method. It should be mentioned that the use of VAS has been previously reported as an appropriate tool to determine the subjective hunger/satiety sensations of human beings, after consuming a nutritive product [16,17].

### 2.5. ObjectiveHunger/Satiety Analyses

Objective satiety-associated markers were also analyzed, in addition to the previously mentioned subjective responses of the participants.

#### 2.5.1. Glycemia

Capillary blood was used to quantify glycemia using a commercial glucose meter (OneTouch Ultra Mini, Lifescan, Milpitas, CA, USA). Glycemia was graphed, and AUC was calculated using the trapezoidal method.

#### 2.5.2. Hormones

Venous blood samples were collected at time 0 and time 120, centrifuged at 1500× *g* for 10 min, and the recovered serum was used to quantify the concentration of satiety-associated hormones, namely, insulin, glucagon-like peptide-1 (GLP-1), and cholecystokinin (CCK). Specific ELISA-based kits were used, according to the manufacturers’ instructions (AFG Bioscience, Northbrook, IL, USA for insulin and CCK, and Sigma-Aldrich, St. Louis, MO, USA for GLP-1). Results for all hormones were expressed as pg/mL of serum.

#### 2.5.3. Antioxidant Capacity

Serum antioxidant capacity was quantified at time 0 and time 120, according to the oxygen radical absorbance capacity (ORAC) assay as previously reported [18]. For this, 25 μL of serum (or 75 mM pH 7.4 phosphate buffer as a control) and 150 μL of 10 nM fluorescein were mixed in a microplate well; 25 μL of 240 mM 2,2′-azobis(2-amidinopropane) dihydrochloride (AAPH) was then added. The plate was incubated for 15 min at 37 °C, and fluorescence was read every 90 s at 485 and 529 nm in a microplate reader, for a total of 90 min. Results were expressed as μg Trolox equivalents per mL (μg TE/mL) of serum, as determined with a Trolox standard curve.

### 2.6. Statistical Analysis

A Student’s *t*-test was used to determine statistical differences for each variable analyzed, according to the NCSS 2021 software. Results are shown as mean ± standard error of the mean (SEM), and were considered significant when *p* < 0.05.

## 3. Results and Discussion

### 3.1. Participants’ Anthropometric Characteristics

Eighteen adult consumers participated in the present study, sixteen women and two men. They were young, of normal weight, and apparently healthy. Their overall anthropometric characteristics are shown in Table 1.

### 3.2. VAS

The participants consumed control chips, followed by 10% AP-supplemented corn chips a week after; their subjective hunger/satiety sensations are shown in Figure 2.

The participants’ hunger was initially high, according to values of nearly 7 (Figure 2A, 95% confidence interval, [CI]: 5.5, 7.7 for the control and 5.0, 6.9 for the AP-supplemented samples), since they had not consumed food overnight; it then decreased to approximately 4 (95% CI: 3.1, 5.5 for the control and 3.4, 5.5 for the AP-supplemented samples) 15 min after consuming the corn chips. A progressive increase in hunger can be seen throughout the remaining testing period (up to 120 min); their final responses were nearly identical to their initial ones (95% CI: 5.2, 6.9 for the control and 5.8, 7.1 for the AP-supplemented samples). No significant differences were found between the control and the AP-supplemented samples at any time.

Prospective food consumption (Figure 2B) followed a similar pattern to hunger, since they began with high responses of approximately 7 (95% CI: 5.7, 7.6 for the control and 6.1, 8.0 for the AP-supplemented samples), which slightly decreased 15 min after consuming the samples (95% CI: 4.6, 6.4 for the control and 4.1, 6.1 for the AP-supplemented samples). Their responses also ended with nearly identical values to the initial ones (95% CI: 6.2, 7.9 for the control and 5.8, 7.2 for the AP-supplemented samples). No significant differences were found between the control and the AP-supplemented samples at any time.

Significant differences were found in the satiety responses (Figure 2C) between the AP-supplemented samples and the control. It is apparent that they began the experiment with very low values of approximately 2 (95% CI: 1.2, 2.6 for the control and 1.2, 3.0 for the AP-supplemented samples), which indicated minimal-to-no satiety sensation. A total of 15 min after consuming the control samples, their satiety increased to approximately 4.5 (95% CI: 3.5, 5.8), while it increased to approximately 7 in response to consuming the AP-supplemented samples (95% CI: 6.1, 8.1). The control maintained similar values of approximately 4 throughout the experimental period, while the AP-supplemented samples induced significantly higher satiety sensations during the first 60 min; the curves converged and ended at similar values thereafter (95% CI: 2.8, 4.6 for the control and 3.5, 5.1 for the AP-supplemented samples).

Fullness responses (Figure 2D) show a similar pattern to those of satiety. Participants began with a low fullness sensation of slightly below 2 (95% CI: 0.8, 2.4 for the control and 0.9, 1.9 for the AP-supplemented samples), which increased to 4 in response to consuming the control (95% CI: 3.3, 5.8). Their fullness sensation increased to approximately 7 when consuming the AP-supplemented samples (95% CI: 6.1, 7.4), which was significantly higher throughout the first 30 min of the experiment (95% CI: 3.1, 5.3 for the control and 5.5, 6.4 for the AP-supplemented samples), followed by similar values to those of the control at 60 min and after (95% CI: 2.5, 4.1 for the control and 3.5, 4.9 for the AP-supplemented samples).

It is therefore apparent that the addition of 10% AP to corn chips increased the participants’ subjective satiety and fullness responses, without having an impact on their hunger sensation or prospective food consumption during a 120 min postprandial period.

The AUC values of the aforementioned VAS responses are shown in Figure 3.

The observed pattern is comparable to the one reported for VAS, where statistically similar values can be seen for hunger (95% CI: 539, 724 for the control and 537, 678 for the AP-supplemented samples) and prospective food consumption (95% CI: 629, 805 for the control and 638, 791 for the AP-supplemented samples). Regarding satiety (95% CI: 393, 601 for the control and 546, 727 for the AP-supplemented samples) and fullness (95% CI: 353, 548 for the control and 515, 649 for the AP-supplemented samples), their AUC values were significantly higher for the AP-supplemented samples, as compared to the control. This suggests that both the VAS responses throughout time and the corresponding AUC show congruent results, where the addition of 10% AP to corn chips increased the participants’ subjective satiety and fullness, while exerting no changes on hunger and prospective food consumption.

The analysis of subjective variables like the ones reported in the present work is useful to determine how individuals actually perceive their current state of hunger/satiety. Such sensations are continuously varying and fluctuate throughout the day as a result of integrating multiple intrinsic and extrinsic inputs [19]. One variable that has shown a significant correlation to hunger/satiety is gastric emptying, where slower emptying results in decreased hunger and vice versa [20]. Gastric distention directly relays information to the central nervous system about the presence of food in the stomach, such that satiation/satiety are promoted and hunger is inhibited [21].

The data reported in the present study suggest that, even while the participants’ hunger and prospective food consumption remained unaffected, they did report feeling a higher sensation of satiety and fullness. This may be evidence that the composition of AP resulted in a stronger sensation of “having a full stomach” as soon as the corn chips were consumed, since the differences were significant during the first 60 (satiety) and 30 (fullness) min post-consumption. This may be potentially attributed to two kinds of molecules, fiber and phenolic compounds. Fiber is known as a satiety-inducing component of food, due in part to delaying gastric emptying and, thus, prolonging fullness and satiety when added to an edible product [22]. Phenolic compounds exert complementary effects to those of fiber, including stimulating the secretion and/or stimulatory action of satiety-associated enteroendocrine signaling molecules [23]. Accordingly, a fiber- and phenolic-containing food is likely to be a stronger satiety inducer, as compared to one that lacks them. In the case of AP phenolics, there is direct evidence that demonstrates their effects on satiety, albeit, in an animal model after consuming them for eight weeks [7].

### 3.3. Glycemia

The participants’ glycemia was measured in parallel to their previously mentioned subjective hunger/satiety sensations. These data were used to determine the AUC for glycemia, and the results are shown in Figure 4.

Regarding glycemia (Figure 4A), participants began the experiment with values in the range of 90–100 mg/dL with no significant differences (95% CI: 93.8, 100.0 for the control and 86.5, 97.4 for the AP-supplemented samples), as expected for healthy adults after an overnight fast. Glycemia increased 15 min after consuming both samples, with the control reaching significantly higher values of 137 mg/dL (95% CI: 125.8, 147.3), as compared to 118 mg/dL for the AP-supplemented samples (95% CI: 107.1, 128.8). Values for both samples were similar after 30 min (95% CI: 133.0, 153.3 for the control and 123.7, 143.1 for the AP-supplemented samples) and remained similar for the rest of the experimental period.

The corresponding AUC for glycemia is shown in Figure 4B, where it is apparent that, even while there were differences in glycemic responses, the AUC was similar for both samples with values of approximately 15,000 mg × min/dL (95% CI: 14,037, 15,283 for the control and 13,405, 15,233 for the AP-supplemented samples). It appears that the change in glycemia documented 15 min post-consumption was not sufficient to decrease the overall AUC; thus, a stronger change may be required to significantly alter this value.

As consumers demand palatable ready-to-eat foods, processed foods have become increasingly common in everyday diets, even though they are known to have multiple attributes that make them unsuitable for regular consumption, including having a high impact on the consumer’s postprandial glycemia. A food’s impact on this value is indicative of the ease with which its carbohydrates can be digested and absorbed [24]. Foods with a high impact are considered detrimental to health due to them promoting rapid and marked glycemic fluctuations; their acute and regular consumption has been associated with alterations in cardiovascular and hepatic health [25,26], while they are often restricted or eliminated entirely from the diet of diabetic patients in lieu of foods with a lower impact [27]. Foods of vegetable origin are known for having generally low effects on postprandial glycemia, such as avocado, while vegetable-derived byproducts have been recently explored as options to lower it. For example, okra seed byproducts and apple pomace have been studied in muffins and biscuits, respectively, for this purpose, with both of them showing significant reductions in the supplemented products’ glycemic impact [28,29]. Others have also shown that phenolic compounds can potentially contribute to this lowering effect, such as in the case of chickpea and chicory byproducts in bread, further supporting the potential of vegetable byproducts in lowering glycemic impact [30].

### 3.4. Hormones

The concentration of three hormones was measured before and after consuming the corn chips. The concentration of insulin is shown in Figure 5.

Values of approximately 250 pg/mL were recorded before (95% CI: 144.4, 328.0) and after (95% CI: 176.0, 348.2) consuming the control samples; the concentration of insulin was approximately 300 pg/mL before consuming the AP-supplemented samples (95% CI: 233.7, 433.7), and decreased to approximately 250 pg/mL after consuming them (95% CI: 162.6, 309.0) (Figure 5A). No significant differences were found when analyzing the effects of consuming the chips; however, when normalizing the changes after consuming both samples (Figure 5B), it is apparent that the control chips resulted in a 10% increase, while the AP-supplemented samples resulted in an approximately 25% decrease.

The concentration of GLP-1 is shown in Figure 6.

Values of approximately 1600 pg/mL are apparent before (95% CI: 1279, 2284) and after (95% CI: 1088, 2026) consuming the control samples; the concentration of GLP-1 was approximately 2200 pg/mL before consuming the AP-supplemented samples (95% CI: 1217, 3153), and increased to approximately 2500 pg/mL after consuming them (95% CI: 1344, 3599) (Figure 6A). No significant differences were found when analyzing the effects of consuming the chips; however, when normalizing the changes after consuming both samples (Figure 6B), it is apparent that the control chips resulted in a 12% decrease, while the AP-supplemented samples resulted in an approximately 12% increase.

The concentration of CCK is shown in Figure 7.

Values of approximately 50 pg/mL are apparent before (95% CI: 20.6, 75.0) and after (95% CI: 25.6, 84.2) consuming the control samples; the concentration of CCK was approximately 75 pg/mL before consuming the AP-supplemented samples (95% CI: 34.0, 119.2), and increased to approximately 90 pg/mL after consuming them (95% CI: 51.5, 135.9) (Figure 7A). No significant differences were found when analyzing the effects of consuming the chips; however, when normalizing the changes after consuming both samples (Figure 7B), it is apparent that the control chips resulted in a 15% increase, while the AP-supplemented samples resulted in an approximately 23% increase.

The concentrations documented for the hormones analyzed showed no significant differences, although there is an apparent tendency for the AP-supplemented samples to decrease insulin concentration and increase GLP-1 and CCK concentrations. Healthy individuals secrete insulin in response to the postprandial increase in glycemia that occurs after a meal, in order to maintain euglycemic values. Thus, the effect a food has on glycemia is closely related to the consumer’s insulin response [31]. It should also be noted that postprandial glycemia and insulinemia may not necessarily correlate, since the organism’s response to this hormone varies by individual (insulin sensitivity) and, consequently, higher or lower insulin concentrations may be required to normalize glycemia for a given carbohydrate load [32]. Diets rich in vegetable-derived foods tend to have a low glycemic and insulinemic impact, as documented in healthy [33], diabetic [34], and obese [35] consumers. The use of vegetable byproducts has also been considered for this purpose, in order to validate their potential as health-promoting ingredients [36]. The present results suggest that decreasing the corn chips’ glycemic impact in response to the presence of AP (as previously discussed), may result in a lower but non-significant change (*p* > 0.05) in postprandial insulinemia; further experiments are required to confirm the effects of AP on insulin secretion and action.

In contrast to insulin, the concentration of GLP-1 and CCK tended to increase after consuming AP-supplemented samples, although these changes were also not significant (*p* > 0.05). GLP-1 is closely associated with insulin, since one of its main physiological functions is to promote the glycemic-lowering effect of insulin (i.e., it induces insulin sensitivity) [37], in addition to promoting satiety [38]. CCK is also key for satiety sensations, since it is known to exert it directly [39]. Moreover, CCK receptor activation has been shown to be closely related to the satiety-exerting effects of GLP-1 [40]. Taken together, the tendency of AP-supplemented samples to decrease insulin and increase GLP-1 and CCK concentration suggests a stronger insulin response and satiety, as observed in the participants’ subjective satiety responses and the decreased glycemia of the samples. Nevertheless, it must be stressed that these changes were non-significant tendencies, which suggests the need for additional experiments to fully validate the relationship between AP, hunger/satiety, and serum hormone concentrations, and the mechanisms of action of altering a hunger/satiety sensation may involve multiple other mediators, in addition to the hormones analyzed.

### 3.5. Antioxidant Capacity

Serum antioxidant capacity was measured before and after consuming the corn chips according to the ORAC method; results are shown in Figure 8.

Values of approximately 3200 μg TE/mL are apparent before consuming the control samples (95% CI: 3036, 3386), which decreased significantly to approximately 1300 μg TE/mL (95% CI: 1038, 1620). In contrast, the antioxidant capacity was approximately 2200 μg TE/mL before consuming the AP-supplemented samples (95% CI: 1794, 2554), and significantly decreased to approximately 1900 μg TE/mL (95% CI: 1359, 2391) (Figure 8A). When normalizing the changes after consuming both samples (Figure 8B), it is apparent that the control chips resulted in a significant decrease of approximately 60%, while the AP-supplemented samples resulted in an approximately 15% decrease. This suggests that a postprandial decrease in serum antioxidant capacity was significantly mitigated when consuming AP-supplemented corn chips, as compared to the control.

Postprandial changes in serum antioxidant capacity are known to occur in response to the macro- and micronutrient content of a meal. For example, a Western diet rich in carbohydrates has been shown to decrease antioxidant capacity, potentially associated with prolonged glycemic and insulinemic peaks, while a Mediterranean diet shows a lower impact and may protect against oxidative stress [41]. Such benefits may be due to the higher plant-derived bioactives present in the Mediterranean diet (which is rich in nuts, fish, healthy fats, and other health-promoting foods) such as phenolic compounds [42], since others have reported that plant-based meals mitigate postprandial decreases in antioxidant capacity due to the presence of these molecules [43]. It is also possible that differences in the fatty acid profile of the samples could have impacted the analyzed variables [44], particularly due to the decreased omega-6/omega-3 ratio of the supplemented samples, although this was not specifically considered and remains an open question for future works. According to this information, it is apparent that the effect observed in the present study was a decrease in antioxidant capacity when the control was consumed, and an attenuation of said decrease when the AP-supplemented samples were consumed. These positive effects documented on the consumers’ postprandial antioxidant capacity may be attributed to the bioactive profile of AP, as well as its previously mentioned lower glycemic impact, which together synergize to induce a lower pro-oxidant burden. Consuming avocado-containing diets for eight weeks has been shown to have a favorable effect on antioxidant capacity [45], while the effects of carotenoids from fruit byproducts [46], grape pomace [47], and others have been explored for similar purposes. The present study adds knowledge to the existing literature, regarding the postprandial antioxidant effects of consuming avocado byproducts as a potential health-promoting ingredient. Nevertheless, it should also be mentioned that a systemic antioxidant effect cannot be confirmed or denied with the present data, and further studies are required to determine the full impact of AP on this variable.

### 3.6. Strengths and Limitations

The present work has some notable strengths and limitations. Some of its strengths include the careful selection of participants and their compliance with the experimental protocol. The combined analysis of subjective responses with objective serum biomarkers is also a strength. The use of industry-derived AP, as compared to samples generated under carefully controlled laboratory conditions, further supports its validity.

Some limitations include the population analyzed, which consisted of a limited number of young, healthy, mostly female participants. Although the present results were derived from a pilot study, having a low number of participants is a limitation that could be considered for future studies, in order to improve the robustness of the findings.

Although men and women were equally invited to participate, more women fulfilled the specified criteria of not consuming anything that could interfere with the study (supplements, pharmaceuticals, etc.), not having any diseases that could impact their hunger/satiety, being of good health overall, as well as being interested in taking part in the study. Thus, an imbalanced population of mostly women is another aspect that could be considered for future works. Having a 50/50 male/female population, or as close to it as possible, is likely to provide additional information and make the findings more generalizable.

Hunger/satiety hormones, hedonic hunger, and cravings, are known to fluctuate during the phases of the menstrual cycle [48], and may also be influenced by the use of hormonal contraceptives [49]. These variations were unaccounted for in the present study, given that the menstrual cycle phase of the participants was not homogenized, nor were they asked about their contraceptive use. These hormonal aspects could also be taken into consideration for future studies in order to improve the accuracy of the findings. The results should therefore be interpreted with caution for older or mainly male populations with health conditions that may have an impact on their hunger/satiety sensations, hormone concentrations, or antioxidant capacity (such as insulin resistance, diabetes, obesity, or others).

Participants were not informed which sample was consumed on each visit, and were instructed not to discuss anything related to the samples or their subjective perceptions with each other during the experimental period; however, the chips have some organoleptic differences that could have been unintentionally perceived by the participants, and potentially influence their responses. The sequence of treatment administration could be improved, for example, by randomizing the administration sequence, instead of having all participants consume the control first, followed by the AP-supplemented chips. This modification may avoid familiarization effects.

Hunger/satiety hormones like insulin, GLP-1, and CCK analyzed in the present study may fluctuate during brief periods of time; thus, quantifying them only at the initial and final times of the experiment does not allow determination of the potential changes that may have occurred during this period, making it possible that some significant changes (intermediate peaks) were missed. More frequent sampling times could be considered for future studies.

Avocado processing is likely to vary from one industry to another and potentially from one product to another; thus, the results reported herein from a single source of AP may differ according to the AP source.

## 4. Conclusions

The present study reports industrially generated avocado byproducts, known as avocado paste (AP), as an ingredient used to supplement corn chips. Eighteen apparently healthy adult consumers reported significant acute increases in their subjective satiety and fullness sensations in response to consuming 10% AP-supplemented corn chips, effects that were apparently unrelated to the serum concentration of insulin, glucagon-like peptide-1 (GLP-1), and cholecystokinin (CCK). The addition of AP also resulted in a significantly lower glycemia at 15 min. A postprandial decrease in serum antioxidant capacity was documented in response to consuming the control chips, which the AP-supplemented samples were able to significantly mitigate.

Taken together, the data suggest that AP supplementation altered postprandial glycemia, induced subjective satiety, and mitigated a decrease in postprandial antioxidant capacity in apparently healthy adult consumers. Further studies are required to determine the potential long-term effects of utilizing AP as an ingredient in foods meant for human consumption.

## Figures and Tables

**Figure 1:**
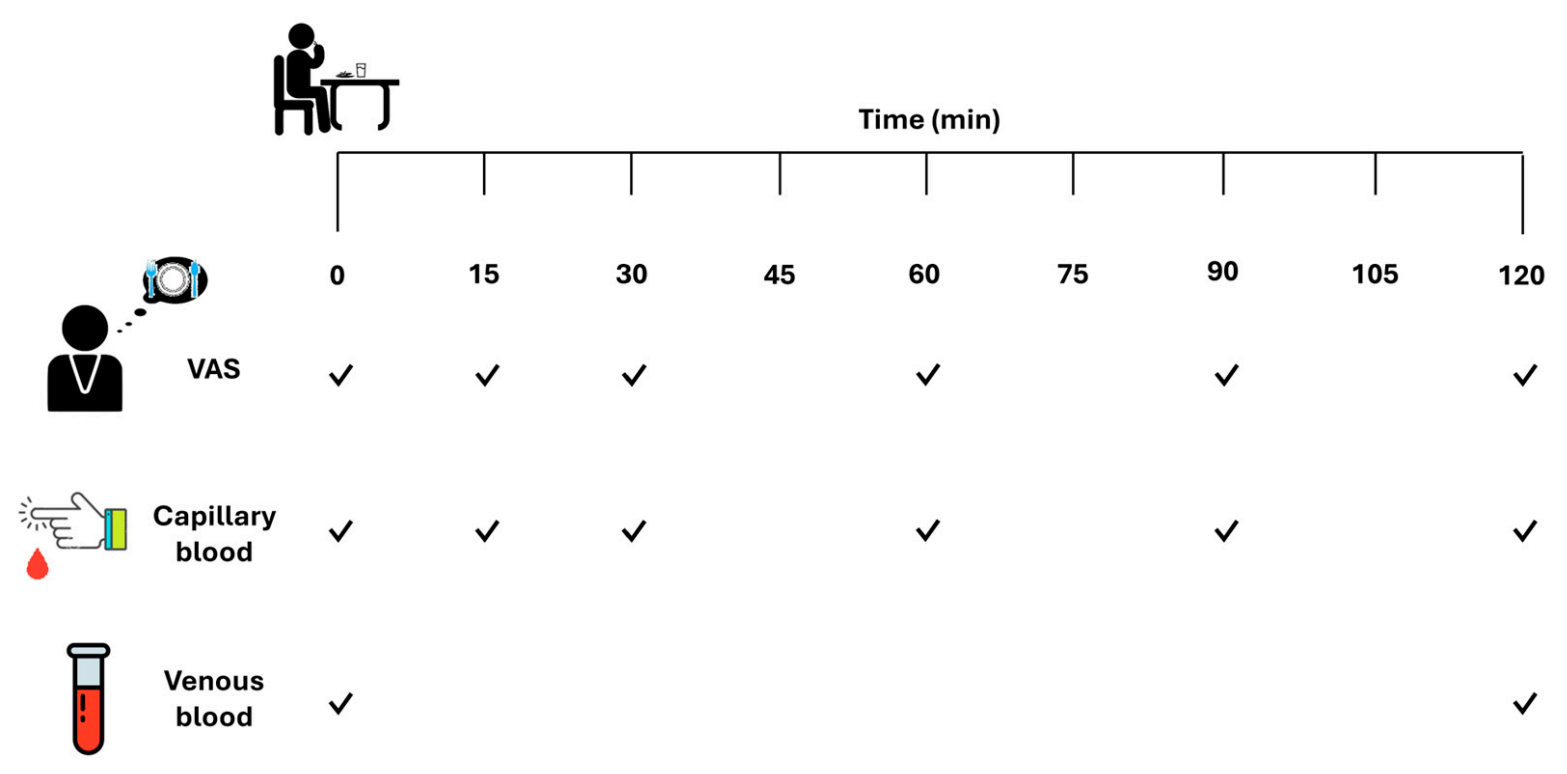
Graphical depiction of the times when each visual analogue scale (VAS) was filled in, and when capillary blood and tubes of venous blood were sampled. A checkmark indicates the time (in min post-consumption) at which a measurement or sample was taken. The same protocol was used for each sample (control and AP-supplemented), which were consumed one week apart.

**Figure 2:**
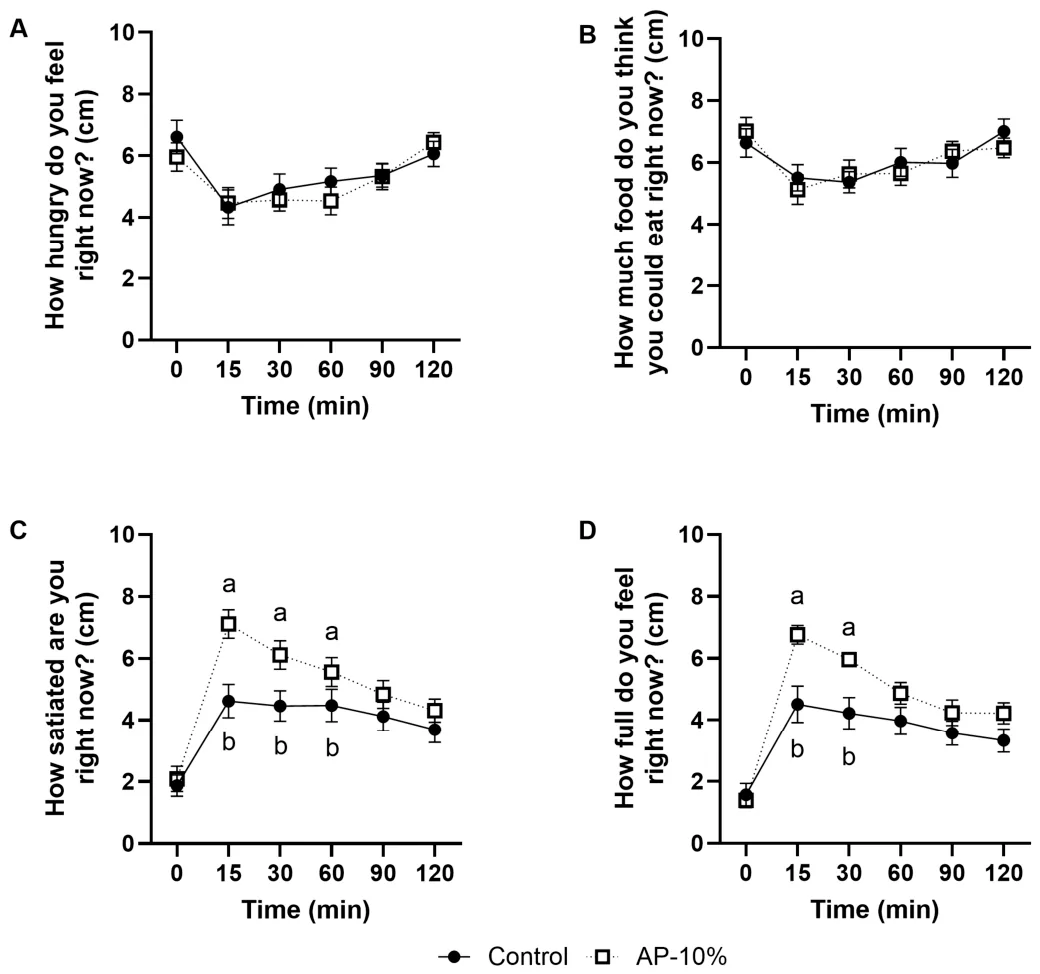
Subjective hunger (**A**), prospective food consumption (**B**), satiety (**C**), and fullness (**D**) responses of eighteen apparently healthy consumers, after consuming the control chips (black circles) or corn chips supplemented with 10% avocado paste (white squares), according to visual analogue scales (VAS). Samples were consumed one week apart. Different lowercase letters indicate significant differences (*p* < 0.05) at each time.

**Figure 3:**
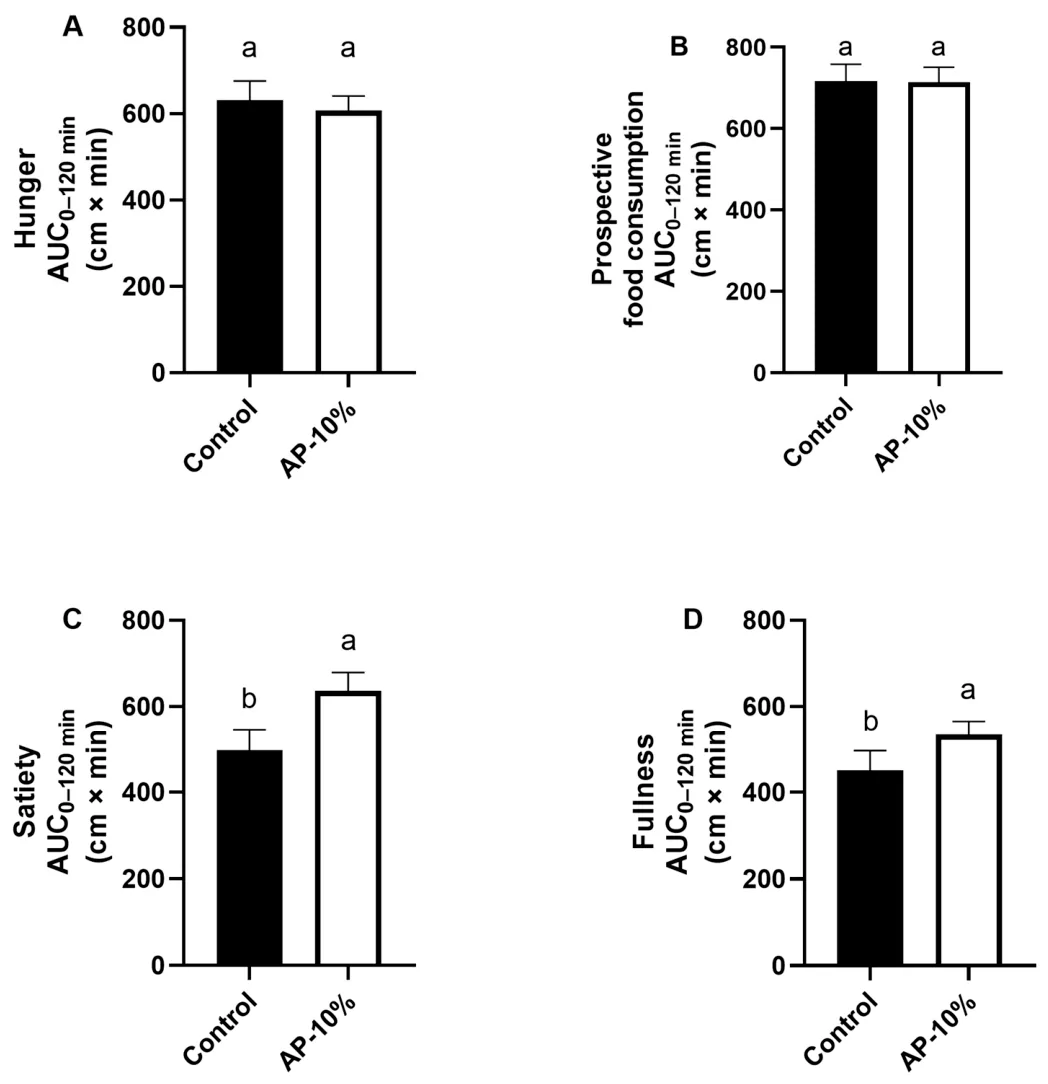
Area under the curve (AUC) of the subjective hunger (**A**), prospective food consumption (**B**), satiety (**C**), and fullness (**D**) responses of eighteen apparently healthy consumers, after consuming a control chip (black bars) or corn chips supplemented with 10% avocado paste (white bars). Samples were consumed one week apart. Different lowercase letters indicate significant differences (*p* < 0.05).

**Figure 4:**
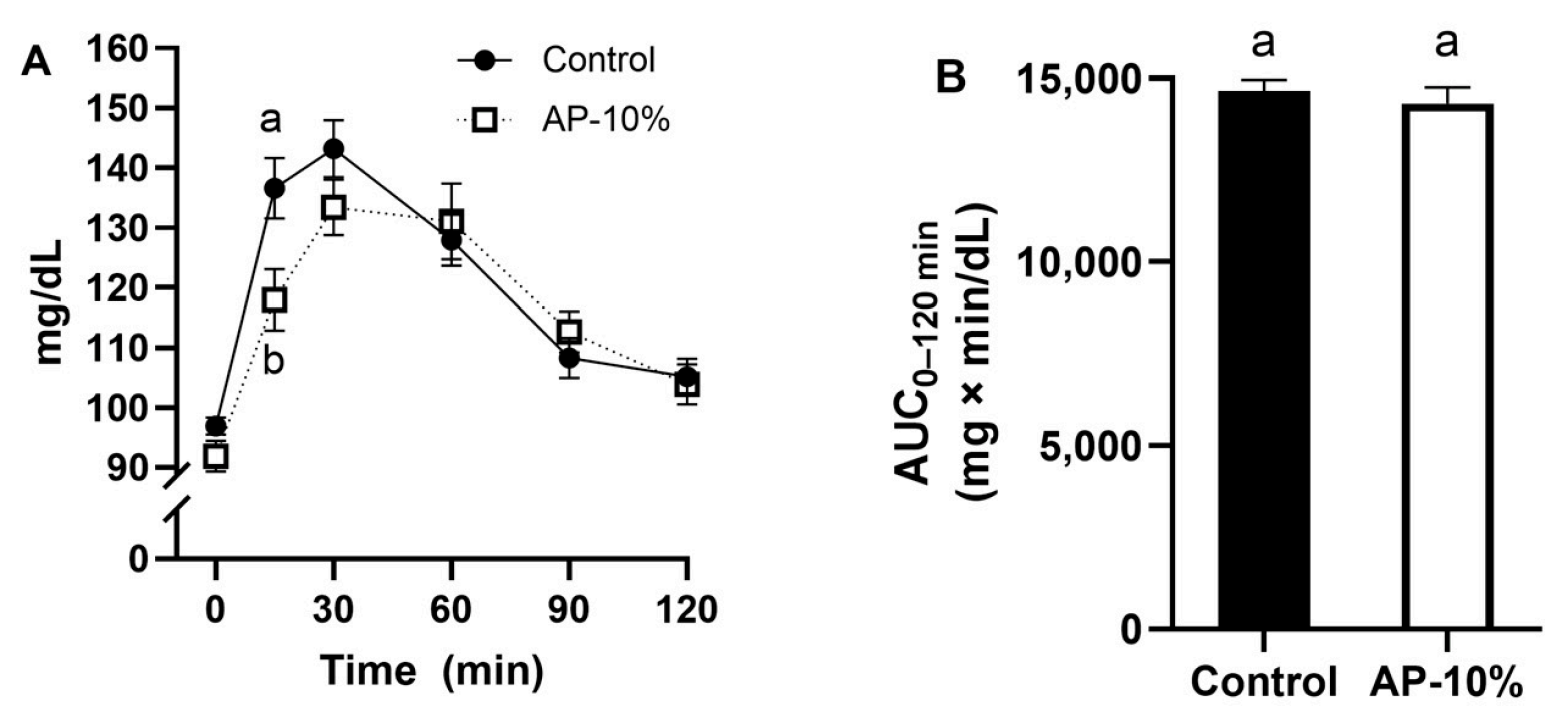
Glycemic responses throughout time (**A**) and area under the curve (AUC) of glycemic responses (**B**) after consuming the control chips (black symbols and bars) or corn chips supplemented with 10% avocado paste (white symbols and bars). Samples were consumed one week apart. Different lowercase letters indicate significant differences (*p* < 0.05).

**Figure 5:**
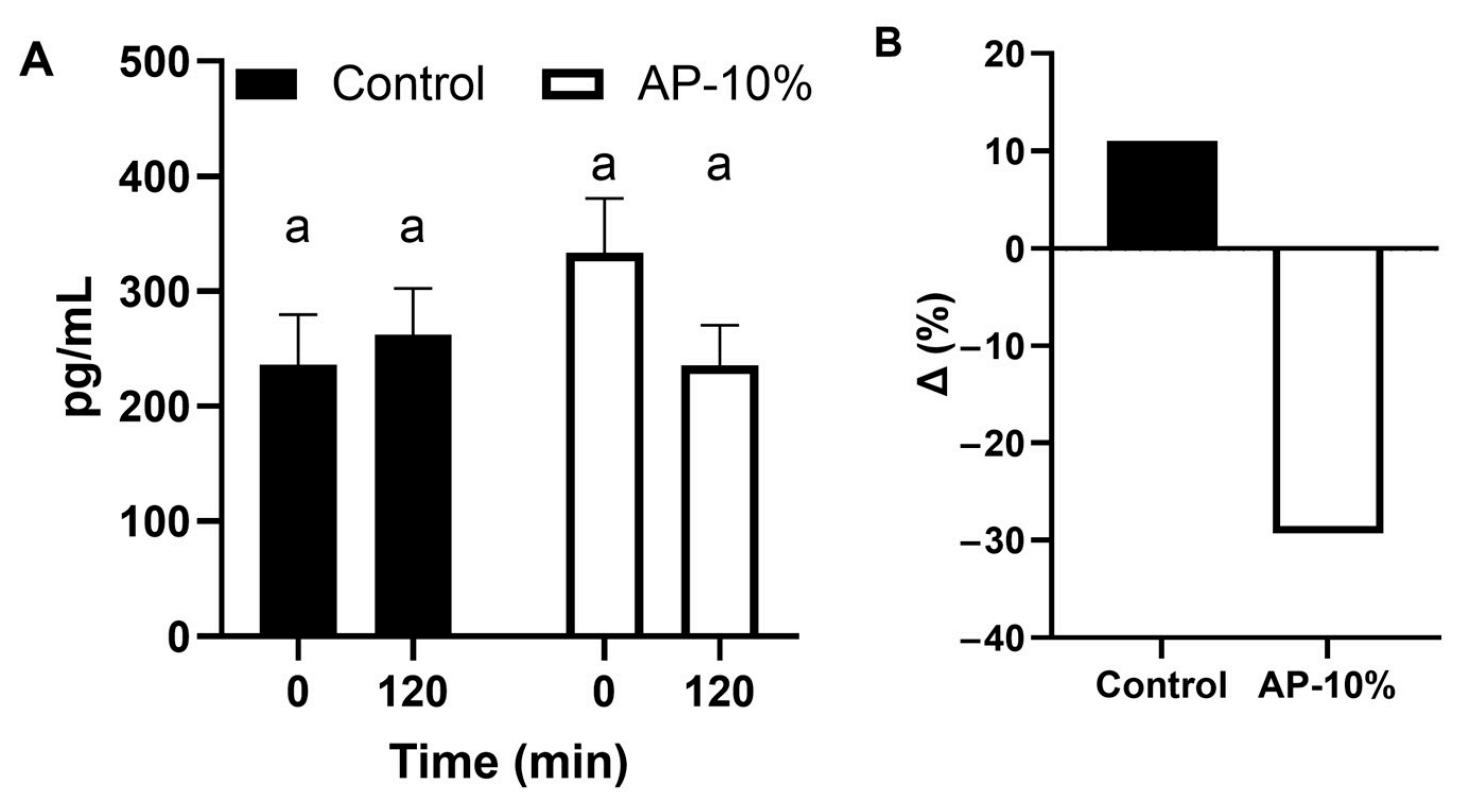
(**A**) Insulin concentrations at time 0 and 120 after consuming the control chips (black bars) or corn chips supplemented with 10% avocado paste (white bars), and (**B**) relative concentration changes (Δ), as compared to time 0. Samples were consumed one week apart. Different lowercase letters indicate significant differences (*p* < 0.05) between the beginning and end of consuming the control or the AP-supplemented samples.

**Figure 6:**
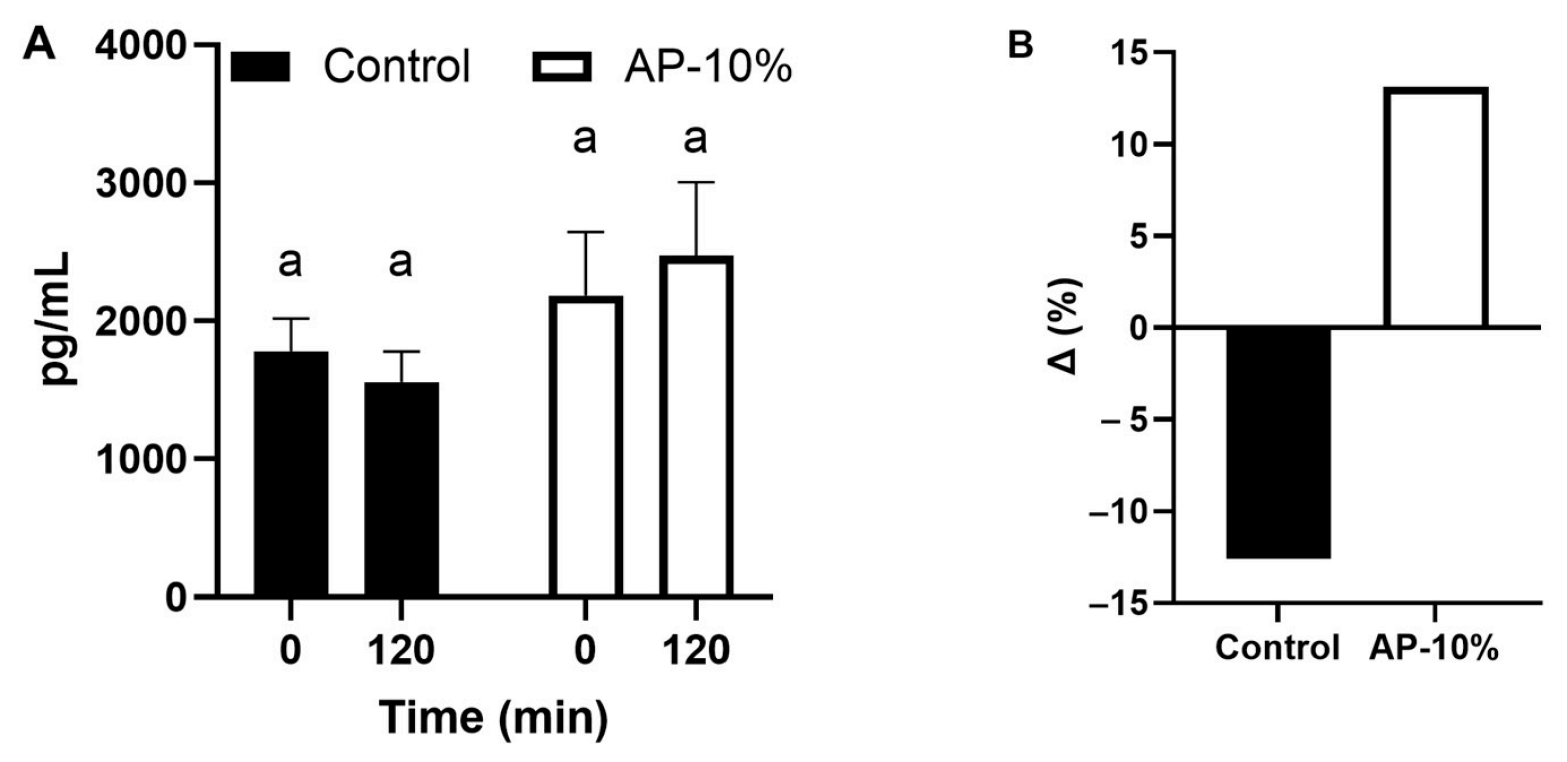
(**A**) Glucagon-like peptide-1 (GLP-1) concentrations at time 0 and 120 after consuming the control chips (black bars) or corn chips supplemented with 10% avocado paste (white bars), and (**B**) relative concentration changes (Δ), as compared to time 0. Samples were consumed one week apart. Different lowercase letters indicate significant differences (*p* < 0.05) between the beginning and end of consuming the control or the AP-supplemented samples.

**Figure 7:**
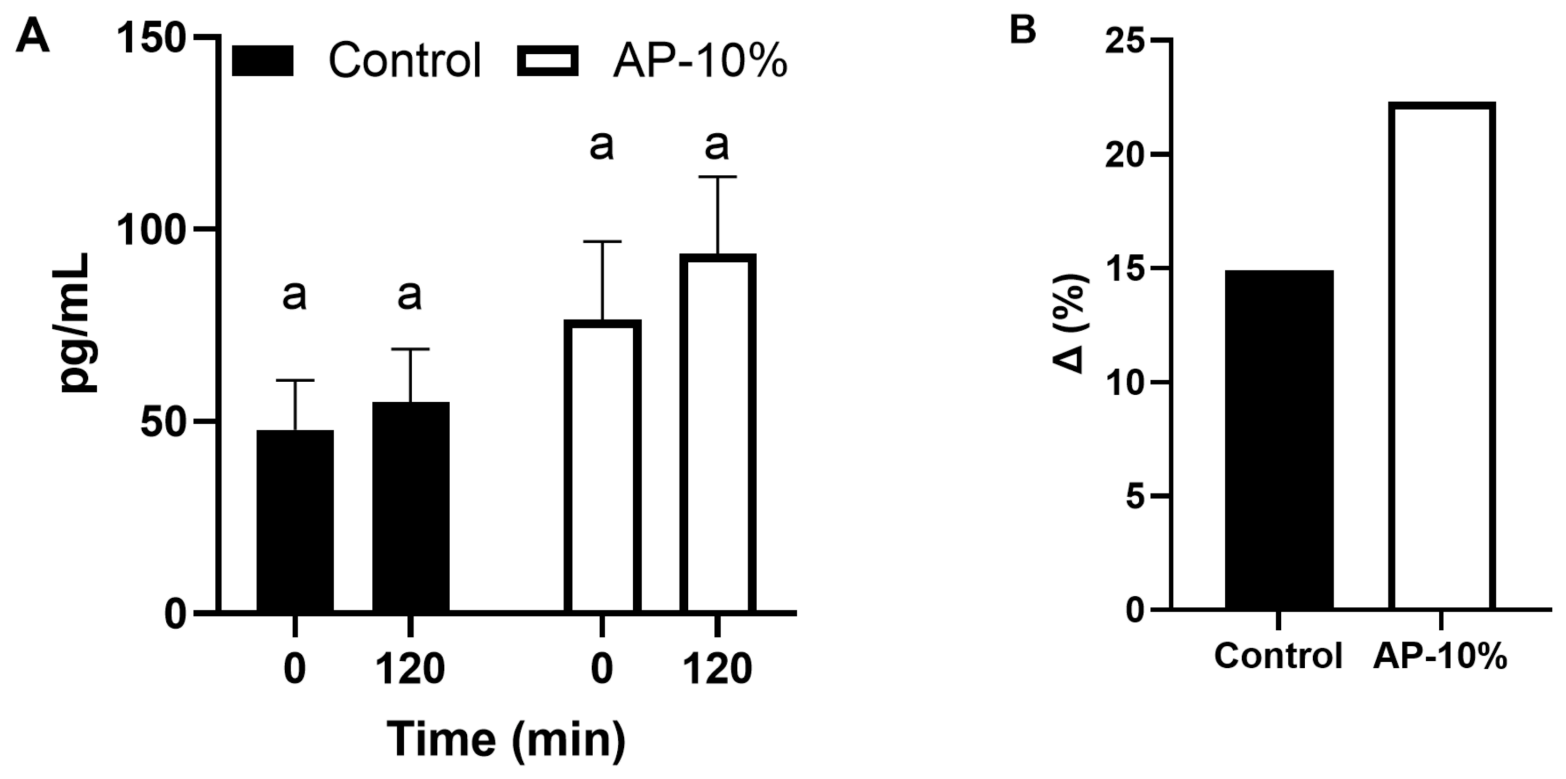
(**A**) Cholecystokinin (CCK) concentrations at time 0 and 120 after consuming a control chip (black bars) or corn chips supplemented with 10% avocado paste (white bars), and (**B**) relative concentration changes (Δ), as compared to time 0. Samples were consumed one week apart. Different lowercase letters indicate significant differences (*p* < 0.05) between the beginning and end of consuming the control or the AP-supplemented samples.

**Figure 8:**
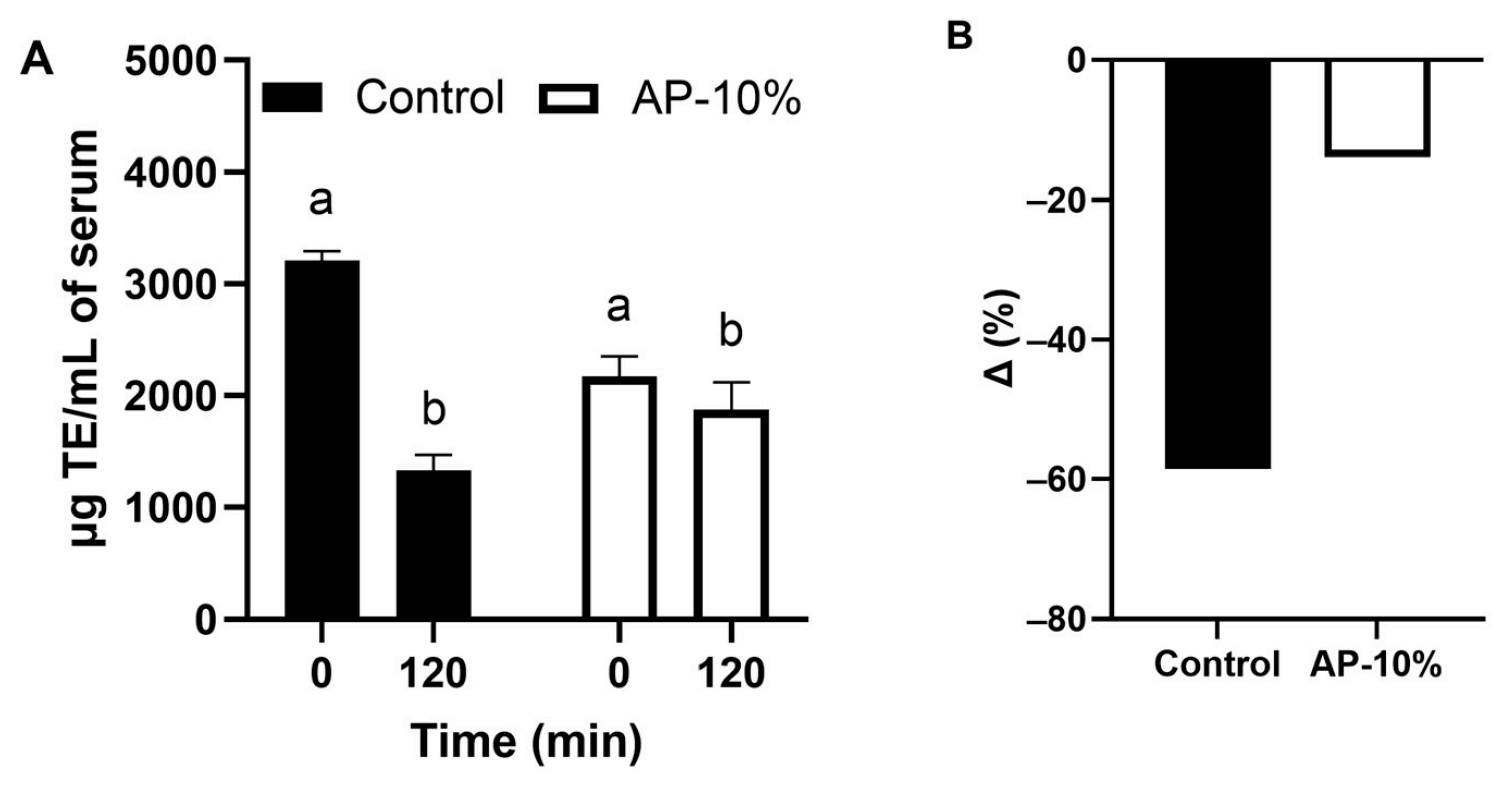
(**A**) Antioxidant capacity (according to the ORAC method) at time 0 and 120 after consuming a control chip (black bars) or corn chips supplemented with 10% avocado paste (white bars), and (**B**) relative antioxidant capacity changes (Δ), as compared to time 0. Samples were consumed one week apart. Different lowercase letters indicate significant differences for each sample (*p* < 0.05) between the beginning and end of consuming the control or the AP-supplemented samples.

**Table 1:** Anthropometric characteristics of the participants of the present study.

Variable	Response
Women	*n* = 16
Men	*n* = 2
Age	22.28 ± 1.61 years
Weight	64.84 ± 12.75 kg
Height	1.66 ± 0.10 m
Body mass index (BMI)	23.53 ± 2.45

## Data Availability

The original contributions presented in this study are included in the article. Further inquiries can be directed to the corresponding author.

## Abbreviations

The following abbreviations are used in this manuscript:

AAPH	2,2′-azobis(2-amidinopropane) dihydrochloride
AP	avocado paste
AUC	area under the curve
BMI	body mass index
CCK	cholecystokinin
GLP-1	glucagon-like peptide-1
ORAC	oxygen radical absorbance capacity
SEM	standard error of the mean
TE	trolox equivalents
VAS	visual analogue scales
